# Unveiling the Pathogenic Influence of TDP‐43 on Tau‐Mediated Neurotoxicity in an Alzheimer’s Disease Mouse Model

**DOI:** 10.1002/alz.088566

**Published:** 2025-01-03

**Authors:** Laura Garcia Toscano, Joshua C. Hincks, Pamela J. McMillan, Misa Baum, Sarah M. Waldherr, Brian C. Kraemer, Nicole F Liachko

**Affiliations:** ^1^ VA Puget Sound GRECC, Seattle, WA USA; ^2^ University of Washington, Seattle, WA USA

## Abstract

**Background:**

Alzheimer’s disease (AD) is the leading cause of dementia and one of the most devastating neurodegenerative diseases. In the last decades, a large proportion of AD patients have been described as having aberrant accumulation of TDP‐43 protein, a well‐established driver of neurodegeneration. This TDP‐43 proteinopathy in AD can co‐occur in neurons with the main hallmarks of the disease, toxic amyloid oligomers and neurofibrillary tangles containing hyperphosphorylated Tau, and correlates with rapid progression and worse prognosis. However, the underlying mechanisms of pathogenic TDP‐43 contributing to AD and its possible involvement as a trigger of neurotoxicity remain unclear.

**Method:**

We aimed to explore the role of TDP‐43 in a mouse model of tau‐mediated toxicity. To this end, we created a new mouse model of tau and TDP‐43 co‐pathology that combines the constitutive transgenic expression of wild‐type human tau with adeno‐associated virus (AAV) driven expression of wild‐type human TDP‐43. Using a stereotaxic device, we performed a unilateral intrahippocampal injection of AAV‐9 carrying the human TDP‐43 cDNA (or green fluorescent protein as a control) into 3‐month‐old transgenic mice expressing the most abundant brain isoform (1N4R) of Tau in neurons. Animals were then perfused 2, 4, and 12 weeks after injection, and brains were collected for immunohistochemistry.

**Result:**

Our results showed that although TDP 43 overexpression was similar in both transgenic and control mice, the presence of hTDP‐43 promoted an increase in phosphorylated tau species. These data suggest a possible interaction between human tau protein and TDP‐43 proteins leading to the spread of phosphorylated Tau species, thus contributing to the pathology in our mouse model of the disease. Additionally, the co‐expression of both TDP‐43 and Tau proteins induced a transient inflammatory state in transgenic animals, sustained 4 weeks after injury. However, 12 weeks after injury, inflammation was resolved, and the presence of phosphorylated Tau species was significantly reduced.

**Conclusion:**

This new mouse model provides a vehicle to explore causes and consequences of co‐pathological tau and TDP‐43. Further studies will be necessary to determine mechanisms underlying TDP‐43 promotion of pathological accumulation of toxic Tau species and neuroinflammation in this experimental model of TDP‐43‐ Tau co‐expression.

